# GLP-1–oestrogen attenuates hyperphagia and protects from beta cell failure in diabetes-prone New Zealand obese (NZO) mice

**DOI:** 10.1007/s00125-014-3478-3

**Published:** 2014-12-20

**Authors:** Robert W. Schwenk, Christian Baumeier, Brian Finan, Oliver Kluth, Christine Brauer, Hans-Georg Joost, Richard D. DiMarchi, Matthias H. Tschöp, Annette Schürmann

**Affiliations:** 1 Department of Experimental Diabetology, German Institute of Human Nutrition Potsdam-Rehbruecke, Arthur-Scheunert-Allee 114-116, 14558 Nuthetal, Germany; 2 German Center for Diabetes Research (DZD), Neuherberg, Germany; 3 Institute for Diabetes and Obesity, Helmholtz Center Munich, German Research Center for Environmental Health (GmbH) and Technical University Munich, Munich, Germany; 4 Department of Chemistry, Indiana University, Bloomington, IN USA

**Keywords:** Beta cells, GLP-1, Liver fat, NZO, Oestrogen, *Pomc*

## Abstract

**Aims/hypothesis:**

Oestrogens have previously been shown to exert beta cell protective, glucose-lowering effects in mouse models. Therefore, the recent development of a glucagon-like peptide-1 (GLP-1)–oestrogen conjugate, which targets oestrogen into cells expressing GLP-1 receptors, offers an opportunity for a cell-specific and enhanced beta cell protection by oestrogen. The purpose of this study was to compare the effects of GLP-1 and GLP-1–oestrogen during beta cell failure under glucolipotoxic conditions.

**Methods:**

Male New Zealand obese (NZO) mice were treated with daily s.c. injections of GLP-1 and GLP-1–oestrogen, respectively. Subsequently, the effects on energy homeostasis and beta cell integrity were measured. In order to clarify the targeting of GLP-1–oestrogen, transcription analyses of oestrogen-responsive genes in distinct tissues as well as microarray analyses in pancreatic islets were performed.

**Results:**

In contrast to GLP-1, GLP-1–oestrogen significantly decreased food intake resulting in a substantial weight reduction, preserved normoglycaemia, increased glucose tolerance and enhanced beta cell protection. Analysis of hypothalamic mRNA profiles revealed elevated expression of *Pomc* and *Leprb*. In livers from GLP-1–oestrogen-treated mice, expression of lipogenic genes was attenuated and hepatic triacylglycerol levels were decreased. In pancreatic islets, GLP-1–oestrogen altered the mRNA expression to a pattern that was similar to that of diabetes-resistant NZO females. However, conventional oestrogen-responsive genes were not different, indicating rather indirect protection of pancreatic beta cells.

**Conclusions/interpretation:**

GLP-1–oestrogen efficiently protects NZO mice against carbohydrate-induced beta cell failure by attenuation of hyperphagia. In this regard, targeted delivery of oestrogen to the hypothalamus by far exceeds the anorexigenic capacity of GLP-1 alone.

**Electronic supplementary material:**

The online version of this article (doi:10.1007/s00125-014-3478-3) contains peer-reviewed but unedited supplementary material, which is available to authorised users.

## Introduction

To compensate for peripheral insulin resistance and glucose intolerance, pancreatic beta cells start to proliferate and increase the biosynthesis and secretion of insulin [1]. The genetic background and environmental factors limit the capacity of this compensation, however, and beta cells eventually fail, leading to type 2 diabetes. In order to prevent this progression, current research focuses on strategies to protect beta cells against the toxic microenvironment produced by circulating carbohydrates and lipids.

Recent data have implicated a beta cell protective role of oestrogen (17β-oestradiol, E2). E2 has been shown to increase insulin biosynthesis via activation of oestrogen receptor α (ERα) [2, 3] and to protect beta cells against toxic lipid intermediates by promoting cell proliferation and inhibition of lipogenesis and apoptosis [4, 5]. Additionally, systemic E2-mediated effects on food intake and energy expenditure also contribute to beta cell protection [6]. In support of these findings, women generally have a lower prevalence of type 2 diabetes than age-matched males, although this changes after menopause [7]. In line with these observations, the EPIC-InterAct study revealed an inverse correlation between age at menopause onset with the risk of developing type 2 diabetes [8]. Despite all these promising findings, oestrogen has not been evaluated as a glucose-lowering drug due to its mitogenic effects in reproductive tissue [9].

Similar to E2, the incretin hormone GLP-1 increases insulin biosynthesis and survival of beta cells, lowers food intake and increases glucose uptake in adipose and muscle tissue [10]. In contrast to more widespread action of E2, GLP-1 action is restricted to tissues presenting the GLP-1 receptor at its cell surfaces. Recently, we showed that hybrid molecules of E2 and GLP-1 (GLP-1–oestrogen) boost the weight lowering effects of GLP-1 in C57BL/6 mice by targeted delivery of oestrogen to the hypothalamus [11]. Consequently, lower doses of the steroid could be used, and the tumourigenic potential of E2 was masked. Although the body weight lowering effects of GLP-1-oestrogen were obvious, the question remained whether the hybrid compound would also be sufficient to protect beta cells under diabetogenic conditions.

Similar to humans, New Zealand obese (NZO) mice develop obesity and insulin resistance as a result of hyperphagia, reduced energy expenditure and insufficient physical activity [12]. The progression from insulin resistance to type 2 diabetes in NZO mice is largely driven by dietary carbohydrates, as carbohydrate-free diets fail to induce diabetes in NZO mice [13]. Taking advantage of this, we previously established a dietary regimen of 13 weeks of carbohydrate-free high-fat diet (to induce obesity and insulin resistance) followed by a carbohydrate-containing high-fat diet that rapidly leads to hyperglycaemia and beta cell destruction [14]. In this study, we used the same model system to investigate the glucose-lowering potential of GLP-1–oestrogen under defined glucolipotoxic conditions.

## Methods

### Animals

**Fig. 1:**
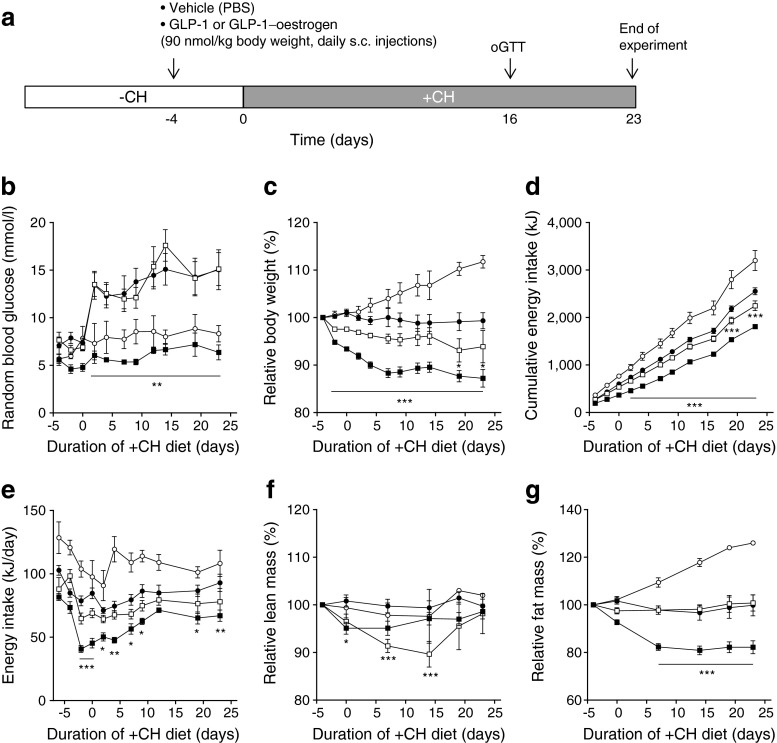
Carbohydrate-induced hyperglycaemia and GLP-1–oestrogen treatment. (**a**) Study design. Random blood glucose (**b**), body weight development (**c**), cumulative energy intake (**d**), energy intake per day (**e**), lean mass (**f**) and fat mass (**g**) were monitored throughout the study. White circles, −CH control; black circles, +CH control; white squares, +CH with GLP-1; black squares, +CH with GLP-1–oestrogen. All data are represented as means ± SEM. Differences compared with the +CH vehicle group were calculated by two-way ANOVA. *p < 0.05, **p < 0.01, ***p < 0.001 vs +CH control group

Male NZO/HIBomDife mice (R. Kluge, German Institute of Human Nutrition, Nuthetal, Germany) were housed in groups of five per cage (type II macrolon) at a temperature of 21 ± 1°C, with a 12 h light–dark cycle (lights on at 06:00 hours). Animals had free access to food and water. All animal experiments were performed in compliance with the German animal protection law (TierSchG). The mice were housed and handled in accordance with the ‘Principles of laboratory animal care’ [15]. The animal welfare committees of the DIfE as well as the local authorities (LUGV, Brandenburg, Germany) approved all animal experiments. At the age of 5 weeks, animals received a carbohydrate-free diet (−CH; 20% (wt/wt) protein and 68% (wt/wt) fat, 29 kJ/g). At the age of 18 weeks, diets were switched to a carbohydrate-containing diet (+CH; 20% (wt/wt) protein, 28% (wt/wt) fat and 40% (wt/wt) carbohydrates, 21 kJ/g) for an additional 23 days (Fig. 1a). Treatment with 90 nmol GLP-1, GLP-1–oestrogen (supplied by R.D. DiMarchi, Indiana University, Bloomington, IN, USA) or oestrogen per kg body weight (daily s.c. injections) started 4 days before the diet switch. Because of the soft texture of the diets, mice had access to wooden gnawing sticks in order to avoid excessive teeth growth.

### OGTT

After an overnight 16 h fasting period, mice received 2 mg glucose per g body weight by oral gavage. At the indicated time points (Fig. 3b) blood glucose and plasma insulin were measured, as previously described [16].

### Immunohistochemistry of pancreatic islets

Pancreatic tissue excised immediately after exsanguination was fixed in 4% (wt/vol.) formaldehyde and embedded in paraffin according to standard procedures. For co-staining of insulin and glucagon, mouse monoclonal anti-insulin (clone K36AC10, Sigma-Aldrich, Munich, Germany) and polyclonal rabbit anti-glucagon (Dako, Hamburg, Germany) antibodies were used. Alexa Fluor 546-labelled anti-rabbit (1:200) and Alexa Fluor 488-labelled anti-mouse (1:200; Invitrogen, Karlsruhe, Germany) were used as secondary antibodies. Nuclei were stained with DAPI.

### Pancreatic insulin content

For detection of the pancreatic insulin content, whole pancreases were homogenised in ice-cold acidic ethanol (0.1 mol/l HCl in 70% ethanol) and incubated for 24 h at 4°C. After centrifugation (16,000×*g*, 10 min) insulin was detected in the supernatant fraction using the Mouse High Range Insulin ELISA (Alpco, Salem, USA).

### Insulin sensitivity index

Whole body insulin sensitivity was calculated after the method of Matsuda and DeFronzo [17]. Briefly, fasting blood glucose (G_0_) and insulin (I_0_) and the mean blood glucose and insulin during OGTT (G and I, respectively) were recorded. Insulin sensitivity index (ISI) was calculated (10,000/square root of [G_0_ × I_0_] × [G × I]).

### Laser micro dissection of hypothalamic nuclei and gene expression analyses

Dissected brains were immediately frozen on dry ice, and RNA was extracted as described previously [11].

### Gene expression analyses in adipose tissue and liver

Total RNA from visceral adipose tissue and liver tissue of mice was extracted, and cDNA synthesis as well as TaqMan gene expression assays were performed as described previously [18].

### Liver histology and triacylglycerol determination

Histological staining of liver connective tissue was performed using a Masson–Goldner staining kit (Merck Millipore, Darmstadt, Germany). For the quantitative determination of triacylglycerol content, livers were homogenised in 10 mmol/l sodium dihydrogen phosphate, 1 mmol/l EDTA, and 1% (vol./vol.) polyoxyethylene-10-tridecyl ether, incubated for 5 min at 37°C, and the triacylglycerols in the supernatant fraction were detected with a commercial kit (RandoxTR-210, Crumlin, UK).

### Islet isolation and transcriptome analysis

Isolation of pancreatic islets was performed by a modified protocol of Gotoh et al [19]. Total islet RNA preparation was performed with the RNAqueous®Micro Kit (Life Technologies, Darmstadt, Germany). RNA integrity was assessed with the RNA6000 nano kit (Agilent, Santa Clara, CA, USA). Microarray analyses were performed by OakLabs (Hennigsdorf, Germany) on a Agilent Mouse 8 × 60 K Chip.

### Statistics

Statistical differences during treatment were determined by two-way ANOVA and Bonferroni posttest. Differences in endpoint measurements were determined by one-way ANOVA and Newman–Keuls Multiple Comparison Tests. Contingency of the expression analyses was calculated by Fisher’s Exact Test. Significance levels were set at *p < 0.05, **p < 0.01 and ***p < 0.001. Data are presented as means ± SEM. For statistical analysis and for graphical presentation GraphPad Prism (5.0; GraphPad Software, San Diego, CA, USA) was used.

## Results

### GLP-1–oestrogen prevents carbohydrate-induced hyperglycaemia

**Fig. 2:**
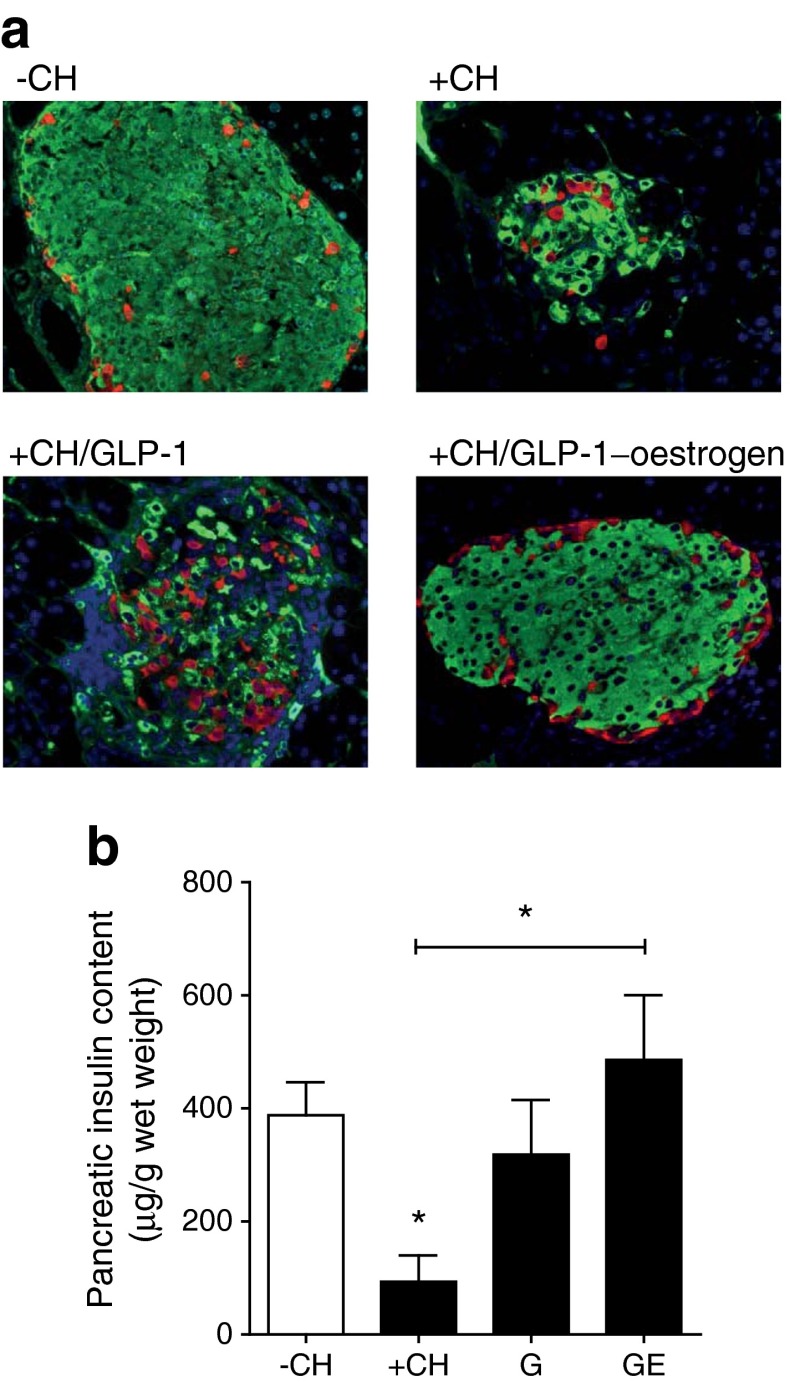
Pancreatic islet integrity. (**a**) Staining of pancreatic slices against insulin (green), glucagon (red) and nuclei (blue). (**b**) Pancreatic insulin content (*n* = 3 animals per group). All data are represented as means ± SEM. Differences compared with the −CH or +CH vehicle group (as indicated) were calculated by Student’s *t* test. *p < 0.05 vs −CH control and +CH control, respectively. G, GLP-1; GE, GLP-1–oestrogen

Male NZO mice were kept on a carbohydrate-free, high-fat diet (−CH) until the age of 18 weeks (Fig. 1a). Due to this dietary regimen, NZO mice become obese and insulin resistant, but are protected from developing diabetes [14]. Afterwards, the diet was changed to a carbohydrate-containing high-fat diet (+CH), which induces a rapid hyperglycaemia and finally beta cell destruction in a synchronised manner [14] (Figs 1b, 2a). GLP-1-treated animals displayed the same increase in blood glucose upon carbohydrate feeding as the vehicle-treated +CH control group (Fig. 1b). In contrast, GLP-1–oestrogen-treated animals exhibited normal blood glucose levels similar to vehicle-treated animals that continued with the –CH diet. Treatment with GLP-1 led to a modest reduction in body weight by 6.1 ± 3.4% from baseline (72 ± 10 g), while GLP-1–oestrogen reduced body weight by 12.8 ± 1.8% (Fig. 1c). The cumulative energy intake of the GLP-1 group only differed at days 19 and 23; animals of the GLP-1–oestrogen group already consumed significantly fewer calories after 2 days of +CH feeding (Fig. 1d). This decreased total amount of consumed calories was largely due to a transient and robust reduction in energy intake during the first 10 days of GLP-1–oestrogen treatment (Fig. 1e). Importantly, the decrease in body weight in the GLP-1 group was due to loss of lean mass (Fig. 1f, g). In contrast, GLP-1–oestrogen-treated animals mainly lost fat mass (Fig. 1f, g).

### Pancreatic islets are protected by GLP-1–oestrogen against carbohydrate-induced destruction

**Fig. 3:**
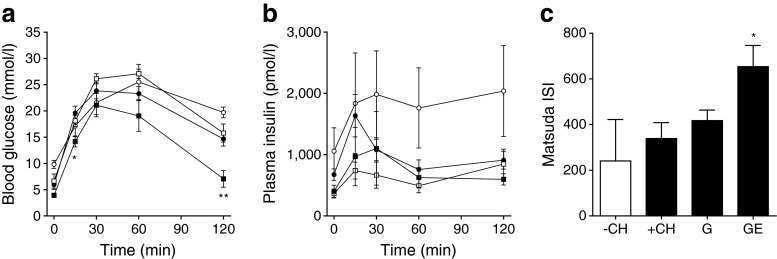
OGTT. (**a**) Blood glucose excursion and (**b**) plasma insulin values (*n* = 5–15 animals per group). (**c**) ISI measured after Matsuda [17]. White circles, −CH control; black circles, +CH control; white squares, +CH with GLP-1 (G); black squares, +CH with GLP-1–oestrogen (GE). All data are represented as means ± SEM. Differences compared with the +CH vehicle group were calculated by two-way ANOVA (**a**, **b**) and one-way ANOVA (**c**), respectively. *p < 0.05, **p < 0.01 vs +CH control group

Histology of the pancreatic islets at the end of the study revealed substantial islet destruction in +CH animals (Fig. 2a). While GLP-1 treatment led to an intermediate phenotype with a clear disruption of normal islet cytoarchitecture, islets from GLP-1–oestrogen-treated animals displayed normal islet morphology (Fig. 2a). To that end, pancreatic insulin content was markedly reduced in response to the carbohydrate intervention (+CH; Fig. 2b). This effect was only partially prevented by GLP-1 (*p* = 0.105); GLP-1–oestrogen resulted in a significant (*p* = 0.034) increase in pancreatic insulin content (Fig. 2b). While GLP-1 treatment alone failed to improve oral glucose tolerance, GLP-1–oestrogen-treated animals displayed improved glucose tolerance (Fig. 3a). Strikingly, fasting glucose levels were highest in −CH control mice, and insulin levels were higher, although not significantly, throughout the glucose tolerance test. This effect could reflect the markedly insulin resistant hepatic glucose output in NZO mice on the −CH diet [13]. Although not significant, both GLP-1 and GLP-1–oestrogen animals showed lower plasma insulin values during OGTT (Fig. 3b). Insulin sensitivity, as measured with the Matsuda index [17], was improved upon treatment with the hybrid compound (Fig. 3c).

### Oestrogen improves islet function but not glucose tolerance

**Fig. 4:**
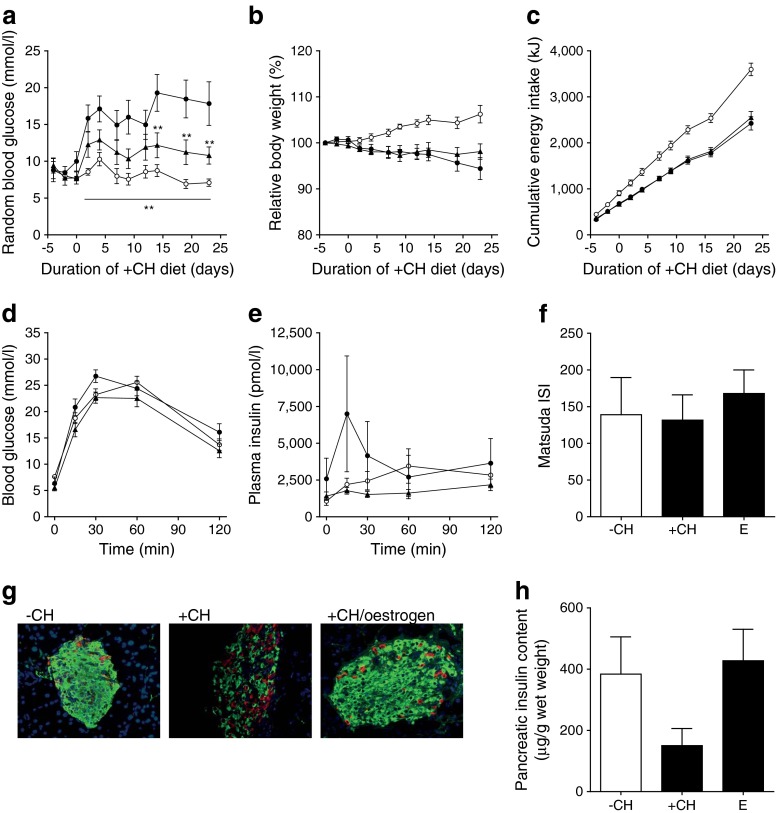
Oestrogen treatment during carbohydrate feeding. Measurements of random blood glucose (**a**), body weight development (**b**) and cumulative energy intake (**c**). Blood glucose excursion (**d**) and plasma insulin (**e**) during OGTT. (**f**) ISI measured after Matsuda [17]. (**g**) Staining of pancreatic slices against insulin (green), glucagon (red) and nuclei (blue). (**h**) Pancreatic insulin content. White circles, −CH control; black circles, +CH control; black triangles, +CH with oestrogen (E). All data are represented as means ± SEM (*n* = 6–10 animals per group). Differences compared with the +CH vehicle group were calculated by two-way ANOVA (**a–e**) and one-way ANOVA (**f**, **h**), respectively. **p < 0.01 vs +CH control group

As effects of GLP-1–oestrogen on diabetes prevention were very much different from that of GLP-1, we performed additional experiments with oestrogen. In oestrogen-treated animals, the carbohydrate-induced rise in blood glucose was reduced but not fully abolished (Fig. 4a). Furthermore, oestrogen treatment did not reduce body weight or energy intake (Fig. 4b, c) and improved neither glucose tolerance nor insulin sensitivity (Fig. 4d–f). Interestingly, pancreatic islets of oestrogen-treated mice displayed largely preserved cytoarchitecture and unaltered insulin content (Fig. 4g, h).

### Reduced food intake is associated with increased anorexigenic signalling

**Fig. 5:**
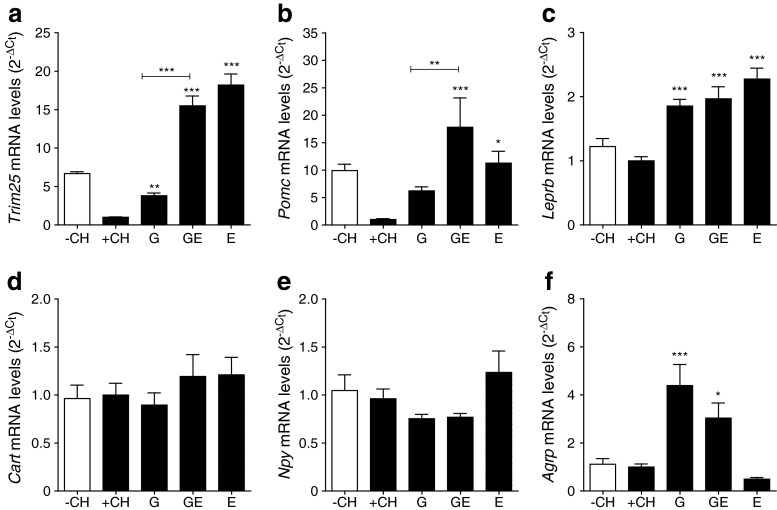
Hypothalamic gene expression. (**a**) *Trim25*, (**b**) *Pomc*, (**c**) *Leprb*, (**d**) *Cart*, (**e**) *Npy* and (**f**) *Agrp* (*n* = 7–11 animals per group). All data are represented as means ± SEM. Differences compared with the +CH vehicle group were calculated by one-way ANOVA. *p < 0.05, **p < 0.01, ***p < 0.001 vs +CH control group. G, GLP-1; GE, GLP-1–oestrogen; E, oestrogen

GLP-1–oestrogen treatment resulted in a transient but substantial reduction in food intake during the first week of treatment (Fig. 1e). Both GLP-1–oestrogen and oestrogen treatment increased the hypothalamic expression of oestrogen-responsive *Trim25* [20] (15.5 ± 1.3- and 18.2 ± 1.4-fold, respectively) in comparison with vehicle-treated +CH control animals, indicating that GLP-1-bound oestrogen is targeted to the hypothalamus (Fig. 5a). Although to a lesser extent, GLP-1 treated animals also displayed elevated *Trim25* expression (3.8 ± 0.9-fold). The carbohydrate challenge suppressed *Pomc* expression, whereas GLP-1 and oestrogen prevented the drop in *Pomc* mRNA levels (Fig. 5b). GLP-1–oestrogen increased *Pomc* expression compared with vehicle-treated and GLP-1-treated animals (17.8 ± 5.4- and 2.9 ± 0.9-fold, respectively; Fig. 5b). Leptin receptor (*Leprb*) expression was increased to a similar extent by all treatments (1.8 ± 0.1-, 2.0 ± 0.2- and 2.3 ± 0.2-fold, respectively; Fig. 5c). Expression of orexigenic *Cart* (also known as *Cartpt*) and *Npy* was not different (Fig. 5d, e). However, expression of orexigenic *Agrp* was elevated with GLP-1 and GLP-1–oestrogen (4.4 ± 0.9- and 3.0 ± 0.6-fold, respectively; Fig. 5e).

### Attenuation of lipogenic pathways in GLP-1–oestrogen-treated mice

**Fig. 6:**
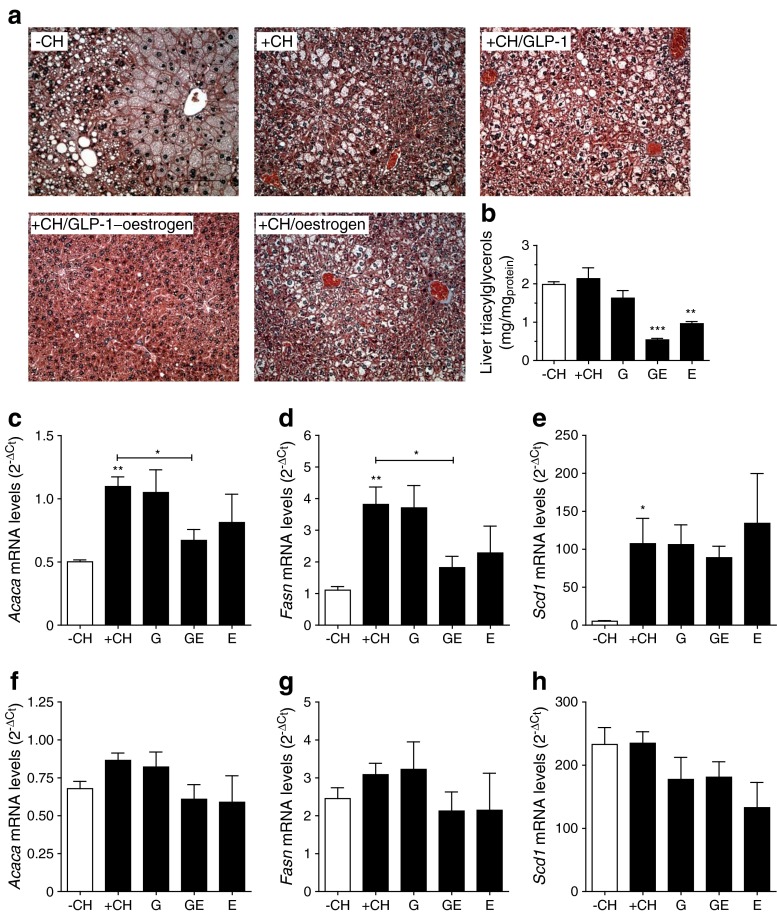
Effects on liver and visceral adipose tissue. (**a**) Masson–Goldner staining of liver sections with cytoskeletal elements and cytoplasma (reddish), nuclei (dark blue) and fibrotic areas (green to blue). (**b**) Hepatic triacylglycerol content. Gene expression of *Acaca*, *Fasn* and *Scd1* in liver (**c**–**e**) and adipose tissue (**f**–**h**) (*n* = 3–6 animals per group). All data are represented as means ± SEM. Differences compared with the +CH vehicle group were calculated by one-way ANOVA. *p < 0.05, **p < 0.01, ***p < 0.001 vs −CH control and +CH control, respectively. G, GLP-1; GE, GLP-1–oestrogen; E, oestrogen

Histological analyses revealed large vacuoles, presumably lipid droplets, in livers of control, GLP-1- and oestrogen-treated mice (Fig. 6a). In contrast, livers of mice that received GLP-1–oestrogen showed substantially improved liver morphology. Accordingly, hepatic triacylglycerol accumulation was attenuated in GLP-1–oestrogen-treated mice, but also oestrogen-treated mice revealed lower triacylglycerol levels (Fig. 6b). Carbohydrate feeding substantially increased hepatic expression of lipogenic *Acaca*, *Fasn* and *Scd1* (Fig. 6c–e). GLP-1–oestrogen reduced expression of *Acaca* and *Fasn* by 39 ± 7.8 and 52 ± 9.3%, respectively. Neither of the treatments altered expression of *Scd1* (Fig. 6c). Importantly, hepatic expression of *Trim25* was not different among the treatment groups (electronic supplementary material [ESM] Fig. 1a). Because loss of fat mass was a major part of the GLP-1–oestrogen phenotype (Fig. 1g), we also investigated the expression of the above mentioned genes in visceral adipose tissue. Although the adipose expression pattern was similar to the hepatic one, no significant differences among the groups were observed (Fig. 6f–h). Also, in adipose tissue, expression of *Trim25* was not different (ESM Fig. 1b), demonstrating that liver and adipose tissue are not a direct target site of action of the GLP-1–oestrogen hybrid.

### GLP-1 and GLP-1–oestrogen treatment affects the transcriptome of pancreatic islets

In order to investigate whether diabetes protection could be due to direct effects of GLP-1–oestrogen on pancreatic islets, we studied the islet transcriptome 2 days after diet switch (6 days after treatment initiation). At this time point, the rise in blood glucose and suppression of Akt signalling is observed in untreated NZO mice [14]. In the islets of GLP-1-treated animals, 120 mRNAs were differentially expressed compared with the islets of the +CH group (signal intensity > 50, log fold change > |1.0| and *p* < 0.05; ESM Table 1). Similarly, in islets of GLP-1–oestrogen mice, 43 mRNAs were differentially expressed (ESM Table 2). Of these mRNAs, 21 were upregulated (Table 1) and 13 were downregulated (Table 2) by GLP-1–oestrogen treatment only, indicating effects specific for the hybrid compound.

**Table 1:** Genes selectively upregulated by GLP-1–oestrogen treatment

Gene symbol	Gene name	Target name	Log_2_ ratio	*p* value
n.d.	n.d.	ENSMUST00000090946	1.09486	0.0402206
*Ddn*	Dendrin	NM_001013741	1.07733	0.0024692
*Uap1l1*	UDP-*N*-acteylglucosamine pyrophosphorylase 1-like 1	NM_001033293	1.07954	0.0140211
*Vmn2r3*	Vomeronasal 2, receptor 3	NM_001104614	1.13616	0.0201833
*C2cd4a*	C2 calcium-dependent domain containing 4A	NM_001163143	1.18812	0.0451038
*Psat1*	Phosphoserine aminotransferase 1	NM_001205339	1.0513	0.00834699
*Lce1k*	Late cornified envelope 1K	NM_001254760	1.43164	0.0408397
*Hemt1*	Haematopoietic cell transcript 1	NM_010416	1.10077	0.0423324
*Olfr48*	Olfactory receptor 48	NM_010990	1.21633	0.0475563
*Dapp1*	Dual adaptor for phosphotyrosine and 3-phosphoinositides 1	NM_011932	1.07411	0.0328786
*Ar*	Androgen receptor	NM_013476	1.58906	0.0127427
*Pvalb*	Parvalbumin	NM_013645	1.0195	0.00177101
*Gcat*	Glycine *C*-acetyltransferase (2-amino-3-ketobutyrate-coenzyme A ligase)	NM_013847	1.08209	0.0187919
*Chst11*	Carbohydrate sulfotransferase 11	NM_021439	1.06272	0.0296596
*Ankrd22*	Ankyrin repeat domain 22	NM_024204	1.00047	0.0168974
*Olfr521*	Olfactory receptor 521	NM_146356	1.76128	0.0398948
*Zfp846*	Zinc finger protein 846	NM_172919	1.03213	0.00931397
*Esyt3*	Extended synaptotagmin-like protein 3	NM_177775	1.31178	0.000172183
*Arl9*	ADP-ribosylation factor-like 9	NM_206935	1.19194	0.0302383
*Pinc*	Pregnancy induced noncoding RNA	NR_003202	1.11694	0.0441702
*Gm11213*	Predicted gene 11213	NR_028584	1.11473	0.0430175

n.d., not determined

**Table 2:** Genes selectively downregulated by GLP-1–oestrogen treatment

Gene symbol	Gene name	Target name	Log_2_ ratio	*p* value
*Trpm1*	Transient receptor potential cation channel, subfamily M, member 1	NM_001039104	−1.3242	0.0442979
*Arrdc4*	Arrestin domain containing 4	NM_001042592	−1.7883	0.0177293
*Fbxo34*	F-box protein 34	NM_001146085	−1.29837	0.000724301
*Ccdc138*	Coiled-coil domain containing 138	NM_001162956	−1.19938	0.0339959
*Slc22a2*	Solute carrier family 22 (organic cation transporter), member 2	NM_013667	−1.67727	0.0484448
*Ramp1*	Receptor (calcitonin) activity modifying protein 1	NM_016894	−1.14732	0.0336212
*Gsto2*	Glutathione S-transferase omega 2	NM_026619	−1.11477	0.0204581
*Hist2h4*	Histone cluster 2, H4	NM_033596	−1.23263	0.0201723
*Trim7*	Tripartite motif-containing 7	NM_053166	−1.04176	0.0152357
*6330416G13Rik*	RIKEN cDNA 6330416G13 gene	NM_144905	−1.09125	0.00117735
*Olfr522*	Olfactory receptor 522	NM_146952	−1.15184	0.0294387
*Scarf2*	Scavenger receptor class F, member 2	NM_153790	−1.64384	0.00309493
*Cox6b2*	Cytochrome c oxidase subunit VIb polypeptide 2	NM_183405	−1.36337	0.00313767

**Fig. 7:**
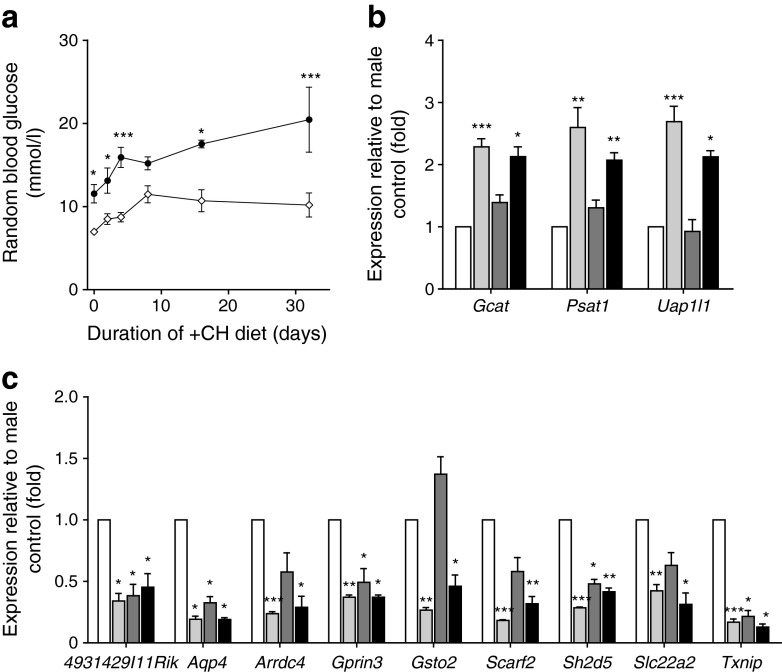
Gene expression in pancreatic islets 2 days after switch to +CH. (**a**) Blood glucose excursion in male vs female NZO mice upon switch to +CH diet. Genes being upregulated (**b**) and downregulated (**c**), respectively, in NZO females and GLP-1–oestrogen-treated males (*n* = 2–3 animals per group). Black circles, +CH males; white diamonds, +CH females. White bars, males; light grey bars, females; dark grey bars, males with GLP-1; black bars, males with GLP-1–oestrogen. Differences in blood glucose were calculated by two-way ANOVA. Expression differences were calculated by Student’s *t* test. *p < 0.05, **p < 0.01, ***p < 0.001 vs control

Oestrogen-specific effects on pancreatic islets could also be the reason for lower diabetes prevalence in female NZO mice [16], which do not display hyperglycaemia upon diet switch (Fig. 7a). In order to narrow down oestrogen-specific effects of the hybrid compound, we performed transcriptome analyses comparing pancreatic islets from male vs female NZO mice 2 days after diet switch. In islets of NZO females, 273 genes were differentially expressed compared with that of male mice (signal intensity > 50, log fold change > |1.0| and *p* < 0.05; ESM Table 3). Twelve (29%) of the genes altered by GLP-1–oestrogen were also differentially expressed in female NZO mice. Contingency analyses exclude an overlap by chance (Fisher’s Exact Test, OR 62.49, *p* = 2.2 × 10^−16^). However, there was also an overlap of 19 genes (16%) between those genes that were altered with GLP-1 treatment and those that differed between females and males (OR 24.14, *p* = 2.2 × 10^−16^). Three genes (*Gcat*, *Psat1* and *Uap1l1*) were enriched in islets of GLP-1–oestrogen-treated male mice and female mice (Fig. 7b). Expression of *Aqp4*, *Gprin3*, *Sh2d5* and *Txnip* was reduced in islets of females, as well as in islets of male mice treated with both GLP-1 and GLP-1–oestrogen (Fig. 7c). Expression of *4931429I11Rik*, *Arrdc4*, *Gsto2*, *Scarf2* and *Slc22a2* was reduced only in islets from females and GLP-1–oestrogen-treated male mice (Fig. 7c).

## Discussion

In this study, we evaluated the potential of oestrogen-coupled GLP-1 to protect beta cell function under glucolipotoxic conditions in diabetes-prone male NZO mice. We show that GLP-1–oestrogen fully prevented the onset of hyperglycaemia and reduced body weight due to a substantially decreased food intake, indicating the hypothalamus to be the main site of GLP-1–oestrogen action. Subsequently, GLP-1–oestrogen protected the mice against carbohydrate-induced beta cell failure, increased glucose tolerance and insulin sensitivity and affected the islet transcriptome. Thus, compared with GLP-1, low-dose GLP-1–oestrogen revealed superior efficacy to preserve beta cell integrity and function under diabetogenic conditions.

The findings of the present study indicate that the combination of GLP-1 and oestrogen in a hybrid molecule possesses a glucose-lowering potential that exceeds the potential of either one of the single molecules. Recently we showed that oestrogen has beta cell protective effects in female NZO mice, as ovariectomised animals displayed elevated blood glucose levels and eventually an increased prevalence of type 2 diabetes compared with sham-operated control mice [16]. Furthermore, treatment of oestrogen-deficient mice with oestrogen resulted in increased protection of pancreatic beta cells against streptozotocin-induced beta cell apoptosis and the collapse of insulin production [5]. In the present study, transcriptome analyses did not indicate direct beta cell protection by GLP-1–oestrogen at the given dose, as neither *Trim25* nor *Acaca*, *Fasn* and *Scd1* were differentially expressed in pancreatic islets of GLP-1–oestrogen-treated mice. The later three lipogenic genes mentioned have previously been shown to be repressed in pancreatic islets of Zucker diabetic fatty rats upon oestrogen treatment [4]. Still, our transcriptome analyses indicated several alterations of the pancreatic expression pattern that could be protective, even as secondary effects of hypothalamic GLP-1–oestrogen action. Particularly, the α-arrestin *Txnip* is well known as a key player in pancreatic beta cell biology, as it is increased in diabetic islets and induces beta cell apoptosis [21, 22]. The GLP-1–oestrogen-mediated suppression of *Txnip* could be mediated via GLP-1 as was shown for exenatide [23]. Additionally, oestrogen-mediated repression of *Txnip* was demonstrated in vitro and in vivo [24]. Still, inhibition of *Txnip* alone was not sufficient to prevent beta cell failure, as seen in GLP-1-treated animals. Interestingly, a second α-arrestin, *Arrdc4*, is suppressed in islets after GLP-1–oestrogen treatment. This effect appears to be mediated by oestrogen, because also females, but not GLP-1-treated males, exhibited this lower expression. Whether *Arrdc4* has similar adverse effects as *Txnip* in beta cells is not known; however, our data suggest that inhibition of both genes in GLP-1–oestrogen-treated mice participates in beta cell protection.

GLP-1-bound oestrogen stimulated anorexigenic signalling that was far more effective than GLP-1 alone. The hybrid compound reaches the brain, as GLP-1–oestrogen treatment increased hypothalamic expression of oestrogen-responsive *Trim25* [20] to a similar extent as oestrogen alone. Nevertheless, induction of *Pomc* expression was clearly highest in GLP-1–oestrogen-treated animals, indicating that oestrogen only affects appetite in NZO mice when combined with GLP-1. Brain-targeted oestrogen not only affects *Pomc* expression but also has been shown to increase the firing rate of *Pomc*-expressing neurons, resulting in substantial reduction in food intake, and subsequently body weight [25]. Therefore, our data are in line with these published observations and suggest that reduced caloric intake via pro-opiomelanocortin (POMC) activation is the major mechanism leading to improved glycaemia in GLP-1–oestrogen-treated NZO mice. Indeed, several studies have proven that caloric restriction is sufficient to improve glucose tolerance and insulin sensitivity in humans and animal models [26, 27]. Especially, liver fat decreases within days upon caloric restriction and contributes to improved glucose homeostasis [28]. In contrast to *Pomc*, expression of anorexigenic *Leprb* was elevated in GLP-1-, GLP-1–oestrogen- and oestrogen-treated mice. In previous studies, NZO mice have been shown to be severely leptin resistant and this leptin resistance might be due to the presence of several polymorphisms in the *Lepr* gene [29, 30]. Therefore, the absence of any anorexigenic signalling in GLP-1- or oestrogen-treated NZO mice could be illustrative of an impaired leptin signalling in NZO mice, despite increased *Lepbr* expression.

GLP-1–oestrogen treatment did not alter *Trim25* expression in liver or visceral adipose tissue. This is opposite to the findings in the hypothalamus and similar to that in pancreatic islets, and could be explained by the lack of GLP-1 receptor expression in these tissues [31, 32]. Interestingly, carbohydrate feeding also suppressed *Trim25* expression in the hypothalamus, but not in the periphery. The mechanism behind this observation, as well as the tissue-specific counter regulation by oestrogen, is not known and data on a metabolic function of *Trim25* are missing. Still, both liver and adipose tissue displayed a substantial reduction in their lipid content, indicating a secondary effect through central actions of GLP-1–oestrogen that ultimately influences whole body metabolism. Furthermore, we observed an inhibition of lipogenic genes in the liver. This is in line with previous studies showing that oestrogen treatment of ovariectomised mice and high-fat diet (HFD)-fed mice resulted in inhibition of lipogenic gene expression in liver and adipose tissue [33, 34]. These published data also indicated enhanced lipolytic response and β-oxidation, which was not assessed in our study and would need further investigation to clarify. However, both studies assumed direct effects of oestrogen on both tissues. The data in the present study suggest that a major part of the oestrogen action is via the central nervous system, as a direct action of GLP-1–oestrogen on liver and adipose tissue are more likely to be excluded. This concept is supported by the finding that specific deletion of hypothalamic ERα is sufficient to reduce whole body energy expenditure and induce hyperphagia in female mice, resulting in obesity and impaired glucose tolerance [35, 36].

In summary, oestrogen-coupled GLP-1 displays superior efficacy in preventing the onset of diet-induced diabetes than GLP-1 alone. In NZO mice, this protective effect is due to central attenuation of hyperphagia, resulting in systemic improvement of glucose tolerance and insulin sensitivity. Therefore, hybrid compounds like GLP-1–oestrogen might be the basis for novel therapeutic options for treating type 2 diabetes mellitus more efficiently.

## Electronic supplementary material

Below is the link to the electronic supplementary material.

ESM Fig. 1 (PDF 15 kb)

ESM Table 1 (PDF 104 kb)

ESM Table 2 (PDF 98 kb)

ESM Table 3 (PDF 79 kb)

## Abbreviations

−CH: Carbohydrate-free high-fat diet
+CH: Carbohydrate-containing high-fat diet
E2: 17β-Oestradiol
ERα: Oestrogen receptor α
GLP-1: Glucagon-like peptide-1
ISI: Insulin sensitivity index
NZO: New Zealand obese
