# Supplementary material for: GLP-1–oestrogen attenuates hyperphagia and protects from beta cell failure in diabetes-prone New Zealand obese (NZO) mice
Source: Diabetologia. 2014 Dec 20;58(3):604–14. doi: 10.1007/s00125-014-3478-3 (PMC4320309; doi:10.1007/s00125-014-3478-3)
Supplement: Supplementary file 1 — (PDF 15 kb) [file 125_2014_3478_MOESM1_ESM.pdf]

ESM Figure 1

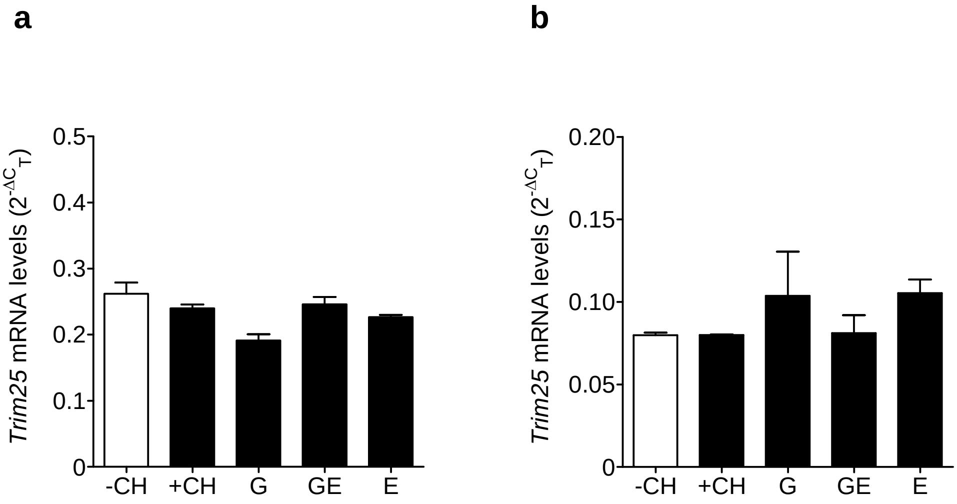

ESM Figure 1 - Expression of estrogen-responsive *Trim25* in (**a**) liver and (**b**) visceral adipose tissue. G, GLP-1; GE, GLP-1-oestrogen; E, oestrogen. All data represented as mean ± SEM. Differences compared to the +CH vehicle group were calculated by one-way ANOVA.
