# Supplementary material for: GLP-1–oestrogen attenuates hyperphagia and protects from beta cell failure in diabetes-prone New Zealand obese (NZO) mice
Source: Diabetologia. 2014 Dec 20;58(3):604–14. doi: 10.1007/s00125-014-3478-3 (PMC4320309; doi:10.1007/s00125-014-3478-3)
Supplement: Supplementary file 2 — (PDF 104 kb) [file 125_2014_3478_MOESM2_ESM.pdf]

ESM Table 1 - Microarray GLP-1

TargetName	+CH1	+CH2	G1	G2	G3	LogRatio	PValue	RefSeqAccession	EntrezGeneID	GeneSymbol
NR_045686	36.7356	40.419	182.708	220.82	172.145	-2.31446	0.00405763	NR_045686	100504104	*Gm16062*
NR_045424	56.6076	43.5702	210.503	131.303	171.192	-1.77143	0.0272525	NR_045424	70491	*5730412P04Rik*
NR_045178	35.9615	34.8247	122.666	101.545	90.8724	-1.56923	0.0103468	NR_045178	73321	*1700042O10Rik*
NR_040439	10.4325	6.45922	68.3856	37.5879	44.3218	-2.56845	0.0416671	NR_040439	69030	*1810006J02Rik*
NR_038009	27.9989	63.9479	111.569	100.797	99.043	-1.17498	0.0273135	NR_038009	69941	*2810408I11Rik*
NR_037679	49.9576	24.9164	414.785	343.305	312.379	-3.25267	0.00417262	NR_037679	321015	*5330439B14Rik*
NR_033578	185.18	124.368	501.224	515.789	418.965	-1.62883	0.00553896	NR_033578	626055	*Gm15645*
NR_028329	40.4811	57.2703	92.7095	116.851	86.0905	-1.01174	0.0353859	NR_028329	654810	*D630032N06Rik*
NR_027924	34.8105	43.7496	99.5316	79.1808	68.9388	-1.07148	0.0371989	NR_027924	68355	*2010204K13Rik*
NR_003513	9731.65	6918.64	19927.4	26318.9	17743.1	-1.35732	0.0334943	NR_003513	66961	*Neat1*
NM_213733	2021.63	2930.52	6034.62	4273.5	4820.8	-1.02622	0.0420977	NM_213733	228961	*Npepl1*
NM_207655	338.42	319.821	922.263	693.319	829.103	-1.308	0.0110159	NM_207655	13649	*Egfr*
NM_207213	580.131	627.409	307.987	287.376	293.063	1.02771	0.000538926	NM_207213	102141	*Snx25*
NM_183183	85.4081	88.6329	58.887	45.1843	25.4287	1.01144	0.0399977	NM_183183	243385	*Gprin3*
NM_181048	58.1844	63.1104	149.981	107.926	108.818	-1.01122	0.0418007	NM_181048	319266	*A130010J15Rik*
NM_178766	98.6452	128.797	246.248	218.992	297.25	-1.16026	0.0213119	NM_178766	319653	*Slc25a40*
NM_177250	102.666	143.131	55.2382	27.7523	57.2703	1.39432	0.0296386	NM_177250	320747	*Lingo4*
NM_176952	260.854	387.779	93.602	119.827	81.3641	1.72266	0.0197137	NM_176952	319582	*6430573F11Rik*
NM_175529	659.529	663.015	1410.16	1263.68	1330.23	-1.01319	0.00115322	NM_175529	243813	*Leng9*
NM_175434	29.7285	32.3102	91.6476	61.1803	69.2246	-1.2547	0.0357032	NM_175434	210027	*Slc35f3*
NM_173763	225.589	282.9	529.981	523.469	534.046	-1.0575	0.00106438	NM_173763	229905	*Ccbl2*
NM_172951	50.8544	46.8386	255.846	219.721	317.276	-2.43575	0.00993086	NM_172951	268534	*Sntg2*
NM_172894	116.754	137.851	274.386	252.289	313.07	-1.13673	0.00809592	NM_172894	243819	*Ppp6r1*
NM_172410	2819.39	2167.78	1147.45	1395.39	999.085	1.07865	0.0192418	NM_172410	71805	*Nup93*
NM_153287	280.168	354.93	1085.79	983.2	839.193	-1.61011	0.00661716	NM_153287	215418	*Csrnp1*
NM_146214	176.897	232.851	410.711	528.003	569.4	-1.29497	0.0193559	NM_146214	234724	*Tat*
NM_144883	760.579	573.909	259.843	357.867	344.237	1.05722	0.0227102	NM_144883	227545	*Proser2*
NM_144817	1520.48	1351.21	547.463	750.187	664.872	1.13416	0.004193	NM_144817	215303	*Camk1g*
NM_144549	634.475	656	1353.97	1362.12	1321.63	-1.06067	0	NM_144549	211770	*Trib1*
NM_133980	26.5361	34.0365	71.8429	65.8437	59.4208	-1.11728	0.00724124	NM_133980	102570	*Slc22a13*
NM_133943	3642.39	3265.59	8261.24	7398.89	5618.39	-1.0381	0.0370891	NM_133943	101502	*Hsd3b7*
NM_133662	146.605	66.9129	245.176	195.744	226.423	-1.05911	0.0456874	NM_133662	15937	*Ier3*
NM_057173	389.719	148.652	608.117	707.29	624.299	-1.2642	0.0310891	NM_057173	109594	*Lmo1*
NM_053107	25.6709	53.1912	77.5793	89.3894	78.3878	-1.05251	0.0337204	NM_053107	93690	*Gpr45*
NM_053078	192.792	169.557	71.5511	88.9409	88.6458	1.1254	0.00329998	NM_053078	27528	*D0H4S114*
NM_033073	3747.7	3092.81	11277.9	9874.34	7889.22	-1.50098	0.016784	NM_033073	110310	*Krt7*
NM_030114	919.389	806.788	1859.85	1893.81	2389.59	-1.24646	0.0133466	NM_030114	67345	*Herc4*
NM_029688	2491.09	3518.46	6944.8	5382.46	7131.1	-1.1101	0.0233035	NM_029688	76650	*Srxn1*
NM_029662	36.6914	43.6935	358.849	311.109	262.384	-2.9509	0.00489803	NM_029662	76574	*Mfsd2a*
NM_028980	93.3039	164.64	335.537	310.206	376.016	-1.40097	0.0100676	NM_028980	74521	*Ppp4r4*
NM_028443	1020.99	1185.64	290.527	532.587	429.149	1.40227	0.00818336	NM_028443	73121	*Fam101a*
NM_028341	54.8102	83.3839	223.678	142.456	169.433	-1.36941	0.0439111	NM_028341	72747	*Ttc39c*
NM_027871	899.975	676.452	2146.78	1598.35	1851.64	-1.24297	0.0164744	NM_027871	71704	*Arhgef3*
NM_027868	793.441	938.589	183.589	207.631	244.714	2.03048	0.00155404	NM_027868	71699	*Slc41a3*
NM_027551	51.702	81.655	33.1505	23.5431	19.9433	1.38414	0.0439664	NM_027551	70788	*Klhl30*
NM_026784	2898.17	2739.7	6661.35	4727.4	5665.44	-1.01194	0.02871	NM_026784	68603	*Pmvk*
NM_026473	800.508	982.065	3182.96	3261.49	2642.32	-1.76485	0.00377534	NM_026473	67951	*Tubb6*
NM_026271	70.8379	51.267	29.5387	24.0115	30.3824	1.12578	0.0234975	NM_026271	67606	*Fibin*
NM_026087	6.41098	7.93888	69.8103	64.3113	53.6858	-3.12518	0.00286507	NM_026087	67315	*Ceacam12*
NM_025685	532.496	841.757	1611.09	1668.06	1252.34	-1.13638	0.0272596	NM_025685	373864	*Col27a1*
NM_025654	1132.96	1045.95	687.831	498.589	413.189	1.03085	0.014799	NM_025654	66599	*Rdm1*
NM_024290	378.715	578.533	972.893	1166.21	915.17	-1.0889	0.0219904	NM_024290	79201	*Tnfrsf23*
NM_023718	213.382	332.768	700.757	505.866	646.262	-1.17744	0.0288835	NM_023718	28254	*Slco1a6*
NM_021455	12628.5	10888.5	5342.59	5665.44	6566.87	1.00515	0.00520186	NM_021455	58805	*Mlxipl*
NM_021385	29.2715	23.8557	71.3078	71.8429	57.8122	-1.33444	0.00753463	NM_021385	58186	*Rad18*
NM_021347	2839.82	1426.75	8298.45	7950.18	6591.1	-1.83544	0.00767255	NM_021347	57911	*Gsdma*
NM_021324	1830.51	1220.78	7590.83	5346.37	4317.5	-1.91453	0.044641	NM_021324	57776	*Ttyh1*
NM_019978	41.7844	26.9843	116.226	86.5959	96.1487	-1.53522	0.0138098	NM_019978	13175	*Dclk1*
NM_019932	147.69	145.917	48.4787	35.2252	23.0165	2.04501	0.00134491	NM_019932	56744	*Pf4*
NM_018824	86.1994	100.133	257.657	284.448	380.275	-1.72252	0.0213201	NM_018824	54338	*Slc23a2*
NM_018811	42.4953	71.0718	132.216	129.024	137.01	-1.22517	0.00632486	NM_018811	54608	*Abhd2*
NM_016879	477.058	622.368	1330.23	1335.15	1001.39	-1.15279	0.0216373	NM_016879	53622	*Krt85*
NM_016789	3.82917	5.35182	102.923	84.8429	109.778	-4.43334	0.00223497	NM_016789	53324	*Nptx2*
NM_013912	44.447	105.767	156.361	152.942	155.644	-1.04509	0.0397886	NM_013912	30878	*Apln*
NM_013846	752.989	853.771	2205.05	1595.83	1947.35	-1.254	0.017047	NM_013846	26564	*Ror2*
NM_013807	1965.81	2932.61	11626.7	14267.7	9346.38	-2.2619	0.0156476	NM_013807	12795	*Plk3*
NM_013692	906.79	1175.04	426.332	484.199	576.694	1.07019	0.0177356	NM_013692	21847	*Klf10*
NM_013666	6051.37	5263.01	13508.3	12550.6	9339.21	-1.06056	0.0339561	NM_013666	225742	*St8sia5*
NM_013614	1348.26	741.82	2249.11	2308.94	1918.06	-1.04661	0.0271543	NM_013614	18263	*Odc1*

NM_013498	533.169	621.761	2001.42	1703.62	1996.38	-1.71855	0.00208152	NM_013498	12916	*Crem*
NM_011817	9346.38	8949.38	3602.64	4950.64	4158.68	1.11029	0.00260471	NM_011817	23882	*Gadd45g*
NM_011780	107.837	80.5428	178.474	245.738	199.547	-1.14238	0.0258748	NM_011780	23792	*Adam23*
NM_011267	1929.63	3086.78	619.051	574.348	923.721	1.82951	0.028549	NM_011267	19734	*Rgs16*
NM_011200	798.852	728.291	2849.69	2241.42	2140.01	-1.65842	0.0105404	NM_011200	19243	*Ptp4a1*
NM_011125	202.75	128.594	76.0857	73.4671	80.0288	1.11429	0.0488477	NM_011125	18830	*Pltp*
NM_011075	84.7549	124.57	51.7298	35.4073	28.9894	1.43501	0.0313605	NM_011075	18669	*Abcb1b*
NM_011065	2334.82	2354.9	5452.13	5045.84	3639.7	-1.00701	0.0443931	NM_011065	18626	*Per1*
NM_010831	124.708	226.91	512.371	421.021	556.293	-1.49796	0.0152954	NM_010831	17691	*Sik1*
NM_010755	359.428	232.423	630.418	852.194	658.803	-1.2703	0.0260642	NM_010755	17133	*Maff*
NM_010706	1068.18	670.061	417.618	314.116	347.923	1.27202	0.0451357	NM_010706	16855	*Lgals4*
NM_010517	125.885	159.05	55.9918	37.0634	91.3018	1.21309	0.0430232	NM_010517	16010	*Igfbp4*
NM_009770	8298.45	10795.6	22935.9	15573.3	20866.4	-1.05179	0.0412736	NM_009770	12228	*Btg3*
NM_009700	454.047	277.267	152.056	88.8921	120.012	1.60361	0.0392898	NM_009700	11829	*Aqp4*
NM_009692	2.47462	5.30079	59.178	48.2411	43.8833	-3.69741	0.00438459	NM_009692	11806	*Apoa1*
NM_009630	196.439	177.511	408.89	410.711	524.695	-1.26097	0.0136158	NM_009630	11540	*Adora2a*
NM_009425	298.147	242.629	101.584	108.664	143.857	1.19582	0.0106756	NM_009425	22035	*Tnfsf10*
NM_008871	52.3651	52.9518	208.441	176.578	171.106	-1.81571	0.00306096	NM_008871	18787	*Serpine1*
NM_008764	575.727	345.724	152.431	171.571	152.684	1.53583	0.0393303	NM_008764	18383	*Tnfrsf11b*
NM_008551	728.745	623.524	1742.31	1400.83	1702.73	-1.25641	0.00741596	NM_008551	17164	*Mapkapk2*
NM_008427	67.4959	42.4311	17.6507	25.611	22.8709	1.31808	0.043933	NM_008427	16520	*Kcnj4*
NM_007972	53.7645	47.095	24.8013	18.1183	18.0375	1.31144	0.00423714	NM_007972	14058	*F10*
NM_007948	136.63	126.211	369.457	283.127	250.083	-1.19504	0.0348622	NM_007948	13870	*Ercc1*
NM_007843	177.358	135.565	22.3045	34.9789	45.0272	2.19782	0.00624215	NM_007843	13214	*Defb1*
NM_007836	126.133	209.722	405.498	393.345	447.533	-1.30687	0.00717361	NM_007836	13197	*Gadd45a*
NM_001267707	98.7751	335.852	608.668	639.108	658.13	-1.54767	0.0189824	NM_001267707	108096	*Slco1a5*
NM_001195084	46.5985	67.5255	28.0353	27.9064	27.8312	1.03101	0.0334387	NM_001195084	18828	*Plscr2*
NM_001190870	34.0924	21.8599	65.1224	49.4837	54.8102	-1.01334	0.03176	NM_001190870	57442	*Kcne3*
NM_001167730	66.4753	56.0006	134.474	147.867	127.121	-1.15627	0.00331455	NM_001167730	58186	*Rad18*
NM_001166157	143.622	113.51	497.635	365.702	324.265	-1.62251	0.0297732	NM_001166157	64818	*Krt81*
NM_001166064	71.4343	54.6061	21.967	31.0179	22.2439	1.32949	0.0141081	NM_001166064	214804	*Syde2*
NM_001162904	166.327	183.659	373.365	494.104	489.897	-1.37048	0.0125168	NM_001162904	17245	*Mdm1*
NM_001144855	52.3307	53.4783	21.4415	26.9843	26.1444	1.08975	0.00113812	NM_001144855	68507	*Ppfia4*
NM_001142920	119.129	74.1872	43.4259	41.3623	38.9741	1.22835	0.0460159	NM_001142920	21416	*Tcf7l2*
NM_001111140	281.856	325.298	1221.54	925.559	705.969	-1.64742	0.044204	NM_001111140	278795	*Lrrc10b*
NM_001099631	759.141	666.458	353.728	295.671	381.797	1.05221	0.00445997	NM_001099631	230863	*Sh2d5*
NM_001099302	704.953	729.109	1518.76	1544.63	1266.64	-1.00931	0.00798726	NM_001099302	434797	*Gm5640*
NM_001085390	40.419	49.694	105.752	123.872	100.592	-1.28864	0.00686935	NM_001085390	240672	*Dusp5*
NM_001081212	3443	3091.8	8106.03	7977.86	7195.19	-1.24786	0.00140594	NM_001081212	384783	*Irs2*
NM_001081121	207.321	171.936	96.8684	39.1426	84.4562	1.36757	0.0213511	NM_001081121	70989	*4931429I11Rik*
NM_001080812	392.519	278.455	98.3964	140.026	183.659	1.2537	0.0349955	NM_001080812	234421	*Cib3*
NM_001039939	1444.59	1451.26	707.879	734.315	607.747	1.08337	0.000601016	NM_001039939	228790	*Asxl1*
NM_001039710	142.989	187.963	389.921	281.264	385.319	-1.08964	0.0314041	NM_001039710	67876	*Coq10b*
NM_001039385	13625.7	15181.7	63650.1	50898.9	47491.6	-1.90689	0.00840296	NM_001039385	381677	*Vgf*
NM_001033149	40.3396	40.9342	92.1998	77.499	75.7455	-1.00957	0.00880802	NM_001033149	69480	*Ttc9*
NM_001029842	303.105	283.178	600.657	572.892	591.439	-1.00503	0.000182526	NM_001029842	104681	*Slc16a6*
NM_001024851	116.897	108.568	41.7844	51.4904	43.3488	1.30767	0.000876294	NM_001024851	545554	*Ankrd34a*
NM_001024474	341.914	280.781	8327.45	5658.12	7355.07	-4.51397	0.00663153	NM_001024474	68203	*Diras2*
NM_001009935	3481.85	6158.14	1041.59	627.044	1433.03	2.22095	0.0358749	NM_001009935	56338	*Txnip*
ENSMUST00000135347	121.598	136.743	353.988	298.757	239.241	-1.20278	0.0299696	XR_141500		
ENSMUST00000128242	4.63288	3.02744	94.7155	50.3014	50.4334	-4.08829	0.0489001	XR_168389	100040493	*Gm13429*
