# Supplementary material for: GLP-1–oestrogen attenuates hyperphagia and protects from beta cell failure in diabetes-prone New Zealand obese (NZO) mice
Source: Diabetologia. 2014 Dec 20;58(3):604–14. doi: 10.1007/s00125-014-3478-3 (PMC4320309; doi:10.1007/s00125-014-3478-3)
Supplement: Supplementary file 3 — (PDF 98 kb) [file 125_2014_3478_MOESM3_ESM.pdf]

ESM Table 2 - Microarray GLP-1-oestrogen

TargetName	+CH1	+CH2	GE1	GE2	GE3	LogRatio	PValue	RefSeqAccess	EntrezGeneID	GeneSymbol
NM_001162956	36356.7	48200.1	17347.1	12737	25147.7	1.19938	0.0339959	NM_0011629	76138	*Ccdc138*
NM_033596	8839.17	6330.81	2998.15	3106.68	3578.34	1.23263	0.0201723	NM_033596	97122	*Hist2h4*
NM_001009935	3451.66	6014.42	813.83	668.989	395.565	2.91824	0.0239883	NM_0010099	56338	*Txnip*
NM_013807	1979.11	2928.77	6350.12	6816.76	9406.83	-1.61651	0.028574	NM_013807	12795	*Plk3*
NM_153790	2388.71	2322.78	885.756	474.466	901.328	1.64384	0.00309493	NM_153790	224024	*Scarf2*
NM_001163143	1428.98	1756.55	2736.64	3844.22	4306.8	-1.18812	0.0451038	NM_0011631	244911	*C2cd4a*
NM_001033293	1236.32	768.798	1929.26	2260.86	2166.19	-1.07954	0.0140211	NM_0010332	227620	*Uap1l1*
NM_013847	750.232	1084.15	1694.11	1948.88	2182.38	-1.08209	0.0187919	NM_013847	26912	*Gcat*
NM_001099631	768.301	674.346	302.447	259.83	336.995	1.26685	0.0026341	NM_0010996	230863	*Sh2d5*
NM_021439	670.308	601.384	1122.7	1277.7	1584.2	-1.06272	0.0296596	NM_021439	58250	*Chst11*
NM_026619	549.82	584.67	245.787	181.82	358.194	1.11477	0.0204581	NM_026619	68214	*Gsto2*
NM_001146085	405.587	384.161	145.007	154.909	181.736	1.29837	0.0007243	NM_0011460	78938	*Fbxo34*
NM_144905	383.625	355.769	170.781	191.123	158.653	1.09125	0.00117735	NM_144905	230279	*6330416G13R*
NM_009700	456.797	277.677	60.2237	71.3217	78.5274	2.39079	0.0213845	NM_009700	11829	*Aqp4*
NM_001042592	278.053	360.194	67.215	60.896	149.06	1.7883	0.0177293	NM_0010425	66412	*Arrdc4*
NM_001039104	277.48	340.342	197.202	108.122	64.7875	1.3242	0.0442979	NM_0010391	17364	*Trpm1*
NM_011932	297.387	273.662	473.873	684.538	645.033	-1.07411	0.0328786	NM_011932	26377	*Dapp1*
NM_001205339	309.832	250.931	515.497	624.054	603.634	-1.0513	0.00834699	NM_0012053	107272	*Psat1*
NM_146952	230.155	282.993	90.3088	94.0808	162.026	1.15184	0.0294387	NM_146952	258954	*Olfr522*
NM_001081121	207.858	172.834	85.1283	123.07	50.1503	1.14427	0.0407427	NM_0010811	70989	*4931429I11Ri*
NM_183405	178.5	148.063	63.1531	65.1279	62.1084	1.36337	0.00313767	NM_183405	333182	*Cox6b2*
ENSMUST00000090946	101.99	208.851	372.612	335.175	288.108	-1.09486	0.0402206	XR_140609		
NM_019932	147.723	145.893	42.0833	34.8292	42.2814	1.88558	0	NM_019932	56744	*Pf4*
NM_010517	125.837	159.199	50.1747	37.9401	42.2439	1.71362	0.00497249	NM_010517	16010	*Igfbp4*
NM_016894	131.17	97.3344	68.8013	42.6993	43.2409	1.14732	0.0336212	NM_016894	51801	*Ramp1*
NM_206935	100.052	118.291	217.705	226.418	304.117	-1.19194	0.0302383	NM_206935	384185	*Arl9*
NM_183183	85.0633	88.3812	28.8063	33.4094	33.9445	1.43593	0.00019508	NM_183183	243385	*Gprin3*
NM_013667	99.6866	61.3855	28.5674	35.9528	11.0241	1.67727	0.0484448	NM_013667	20518	*Slc22a2*
NM_013645	68.5945	73.9125	134.265	149.017	150.055	-1.0195	0.00177101	NM_013645	19293	*Pvalb*
NM_053166	73.7084	57.8244	36.3509	31.7703	27.7141	1.04176	0.0152357	NM_053166	94089	*Trim7*
NM_001166064	70.8914	53.8383	26.8956	32.8877	23.0264	1.17589	0.0179515	NM_0011660	214804	*Syde2*
NM_001254760	50.9515	63.8613	127.864	139.276	197.426	-1.43164	0.0408397	NM_0012547	631101	*Lce1k*
NR_028584	53.9223	47.8087	86.8751	110.071	133.509	-1.11473	0.0430175	NR_028584	670833	*Gm11213*
NM_146356	41.2733	45.833	125.974	123.155	193.806	-1.76128	0.0398948	NM_146356	258353	*Olfr521*
NM_013476	28.0015	48.3795	97.4287	117.574	129.69	-1.58906	0.0127427	NM_013476	11835	*Ar*
NM_024204	34.6001	48.686	77.8085	93.909	78.2216	-1.00047	0.0168974	NM_024204	52024	*Ankrd22*
NM_010416	30.9652	45.6153	81.3627	66.8691	98.1308	-1.10077	0.0423324	NM_010416	15202	*Hemt1*
NR_003202	20.7237	52.2786	85.5483	79.5905	72.3591	-1.11694	0.0441702	NR_003202	723792	*Pinc*
NM_172919	28.7835	38.9819	63.2497	75.396	69.2297	-1.03213	0.00931397	NM_172919	244721	*Zfp846*
NM_001013741	25.7049	35.1054	63.3685	64.1827	64.925	-1.07733	0.0024692	NM_0010137	13199	*Ddn*
NM_177775	27.4575	23.6024	64.1827	62.2476	63.7025	-1.31178	0.00017218	NM_177775	272636	*Esyt3*
NM_010990	15.6187	32.395	52.2561	47.9223	67.1646	-1.21633	0.0475563	NM_010990	18347	*Olfr48*
NM_001104614	20.3863	28.9386	45.5195	57.1971	59.9045	-1.13616	0.0201833	NM_0011046	637004	*Vmn2r3*
