# Supplementary material for: GLP-1–oestrogen attenuates hyperphagia and protects from beta cell failure in diabetes-prone New Zealand obese (NZO) mice
Source: Diabetologia. 2014 Dec 20;58(3):604–14. doi: 10.1007/s00125-014-3478-3 (PMC4320309; doi:10.1007/s00125-014-3478-3)
Supplement: Supplementary file 4 — (PDF 79 kb) [file 125_2014_3478_MOESM4_ESM.pdf]

ESM Table 3 - Microarray male vs female NZO

Gene-ID	male	female	lfc	pval
AK015504	404.1765238	182.5078043	-1.14702737	0.000766
AK030337	211.0616978	456.1375263	1.111804074	0.000137
AK032962	34.9958464	78.25742075	1.161043861	0.005025
AK035765	30.260137	102.745299	1.763581962	0.000486
AK037146	125.5068912	25.13774875	-2.319839222	0.000208
AK042906	50.4883204	114.557886	1.182055186	0.014522
AK049648	99.1080998	214.1462498	1.111521538	0.00012
BC040234	94.4553542	38.28381075	-1.302898136	0.000182
BC096461	234.0975482	523.73078	1.161715571	0.000161
ENSMUST00000001667	606.495291	28.4861975	-4.412161483	0.006631
ENSMUST00000028883	29668.45692	12518.59922	-1.244856753	0.000024
ENSMUST00000036858	611.971934	1245.8544	1.025598079	0.008353
ENSMUST00000107162	89.0451424	189.0115415	1.085865515	0.000071
ENSMUST00000140989	789.0350338	254.1058805	-1.634659596	0
ENSMUST00000155906	103.2641926	42.524616	-1.279969965	0.003419
NM_001005423	241.3989034	584.6258085	1.276094396	0.000006
NM_001007462	250.9397404	81.64145575	-1.619967151	0.000094
NM_001009935	8421.962548	1414.027431	-2.574346352	0.000028
NM_001013377	40.9617806	106.6492228	1.380523117	0.002559
NM_001033212	305.6661986	89.1109385	-1.778282583	0.000034
NM_001033227	2367.047179	46.0187725	-5.68472215	0.015823
NM_001033293	783.2136234	2107.764773	1.428236106	0.000141
NM_001033324	73.5636476	348.2655248	2.243122744	0.002389
NM_001033460	408.9431906	959.592725	1.230521779	0.000027
NM_001034878	114.9768862	53.0467255	-1.116008262	0.004235
NM_001040089	217.5996424	43.668304	-2.31701778	0.000502
NM_001040689	738.756922	271.9207743	-1.441913368	0.000009
NM_001042592	400.2931488	95.10622525	-2.073445242	0.000001
NM_001080812	316.0579706	1315.422556	2.057265211	0.000047
NM_001081121	117.7689962	40.118702	-1.553612951	0.013036
NM_001081123	485.3570558	201.8745588	-1.265587358	0.000087
NM_001081278	352.4814176	146.329731	-1.268324279	0.000138
NM_001085376	15861.02597	7040.364221	-1.171764124	0.000258
NM_001099631	1140.321392	325.1910655	-1.81008097	0.000549
NM_001099774	324.4926862	82.817088	-1.970185581	0.000007
NM_001111030	63.193052	134.191477	1.086455194	0.000008
NM_001113548	63.870218	29.777065	-1.100941814	0.015912
NM_001114157	409.7608082	94.4284645	-2.117488288	0.001299
NM_001122733	46.9387822	148.921842	1.665703048	0.000037
NM_001127346	2434.763887	879.811861	-1.468514917	0.000156
NM_001130479	13489.16668	6738.77009	-1.001244015	0.000016
NM_001143765	255.5570612	76.7705355	-1.735020838	0.018208
NM_001145960	273.6244148	65.2092725	-2.069047934	0.000001
NM_001159487	5530.048926	13239.05567	1.25943607	0.00001
NM_001161832	113.0378444	546.6137975	2.273716017	0.000002
NM_001161844	505.9390504	240.1914955	-1.074778526	0.000249
NM_001162934	134.504426	306.503148	1.188248245	0.000035

NM_001163145	31.016031	64.50844075	1.056473863	0.000004
NM_001163728	494.6145038	212.913052	-1.216040153	0.000218
NM_001177753	552.5930544	141.22536	-1.968218251	0.000004
NM_001195298	64.2160486	142.8563565	1.153559432	0.023327
NM_001204233	532.0100808	119.553513	-2.15379706	0.000047
NM_001205339	307.6151742	799.0130365	1.377092371	0.001254
NM_001242941	84.144729	41.1198505	-1.033037882	0.000002
NM_001252292	27.7192266	55.89722125	1.011889561	0.003844
NM_001252569	126.0184608	277.5846878	1.139292894	0.001079
NM_007472	235.8300824	62.6878205	-1.911490683	0.000718
NM_007492	356.8249412	949.2181915	1.41152329	0.000328
NM_007495	70.0046712	23.33323325	-1.585064959	0.010329
NM_007515	63.7177714	368.7133243	2.532731839	0.000269
NM_007634	30.1407166	61.26600125	1.023372974	0.002396
NM_007705	3500.546544	1562.771662	-1.163473189	0.000029
NM_007706	91.5826918	235.5174325	1.362686975	0.000548
NM_007726	10.8958802	84.8503335	2.961137584	0.000019
NM_007918	840.0445698	2475.746923	1.559326067	0.000477
NM_007969	100.1383672	40.2970065	-1.313250261	0.029105
NM_007992	103.5402122	49.472837	-1.065482642	0.002178
NM_007995	27.4499726	79.272798	1.530021189	0.000421
NM_008051	113.2025038	877.4377288	2.954390874	0.000405
NM_008072	56.6378378	21.62239425	-1.389239909	0.002728
NM_008082	362.564804	171.2124138	-1.082451574	0.000191
NM_008107	165.5205266	373.0472988	1.172348422	0.000085
NM_008137	89.7597744	32.15967575	-1.480816189	0.000149
NM_008223	39.4699134	79.241221	1.005497757	0.024184
NM_008245	141.1792404	325.7237213	1.206120826	0.000003
NM_008250	42.5077444	86.216785	1.020243059	0.001778
NM_008331	23.1647718	53.2149255	1.199898473	0.000274
NM_008433	76.5573674	23.70830675	-1.691148591	0.000396
NM_008489	68.8091094	165.8629263	1.269319975	0.000045
NM_008491	117.879796	51.708645	-1.188839064	0.044413
NM_008638	413.7539824	1144.811177	1.468264559	0.004507
NM_008706	56.3506432	114.901871	1.027898307	0.038403
NM_008760	21.9329008	69.68594125	1.667770997	0.011381
NM_008764	559.137927	260.4135348	-1.102399775	0.000037
NM_008770	5212.723467	1804.696366	-1.530281199	0.000073
NM_008791	212.1849384	734.8338628	1.792095858	0.000072
NM_008815	97.1971888	46.62801325	-1.059717628	0.000025
NM_009065	744.1510036	237.8900243	-1.645300629	0.003927
NM_009215	42854.11994	113569.3509	1.406067731	0.000016
NM_009245	188.746686	421.7903138	1.160074651	0.000461
NM_009246	31.0750058	71.67180025	1.205650932	0.000571
NM_009247	111.103403	255.5266228	1.201570605	0.000456
NM_009311	58.56834	7.52458925	-2.960436269	0.000864
NM_009404	47.928233	96.41883525	1.00843925	0.003481
NM_009549	24.0150728	55.59464225	1.211005673	0.000022
NM_009700	364.824852	70.06186325	-2.380502748	0.020822
NM_009704	2.6689586	61.9171985	4.535991365	0.017324

NM_009719	155.8903278	392.3273093	1.331526344	0.000046
NM_009886	164.982472	353.61888	1.099882545	0.000179
NM_009895	312.8749726	976.015641	1.641318008	0.040458
NM_010165	150.085162	65.05077475	-1.206143209	0.001958
NM_010233	633.026754	290.3310888	-1.12456741	0.000214
NM_010257	86.0365764	37.174407	-1.210640389	0
NM_010321	123.934351	355.1196383	1.518729028	0.000003
NM_010415	913.6678858	417.2194368	-1.130863478	0.004631
NM_010479	60.9958686	332.671234	2.447313688	0.016045
NM_010495	117.1247442	260.1602635	1.151354727	0.000252
NM_010573	281.0234376	692.7313775	1.301605565	0.000265
NM_010574	73.0892232	167.2371738	1.194164962	0.000051
NM_010600	109.5539534	48.7567125	-1.167968789	0.000115
NM_010603	43.427064	210.265567	2.27554629	0.00008
NM_010658	191.4314382	447.3124548	1.22445515	0.000365
NM_010739	214.0371552	86.4637695	-1.30769362	0.000614
NM_010846	404.535291	1061.320371	1.391522743	0.001076
NM_010892	32.5238104	85.16910025	1.388833819	0.009762
NM_010923	23.7737254	64.51196075	1.440198671	0.024283
NM_011020	708.1460298	1449.892776	1.033827412	0.000007
NM_011067	17.7842486	58.15764225	1.709368762	0.000553
NM_011267	2341.425377	559.4953983	-2.065188888	0.000457
NM_011270	255.4442096	88.63748175	-1.527019434	0.000084
NM_011281	224.9424262	108.7796903	-1.048146569	0.00002
NM_011575	92.2882274	214.0092885	1.213454885	0.000043
NM_011584	155.071328	392.1011438	1.338293887	0.000028
NM_011611	121.99215	40.0549095	-1.606737327	0.00102
NM_011623	67.3475928	149.6671233	1.15205906	0.019561
NM_011780	75.9242896	36.1089855	-1.072203617	0.002879
NM_011825	28.3009496	167.3313	2.563784966	0.004139
NM_011837	165.7657468	358.7168655	1.113699652	0.000172
NM_011924	426.2988564	933.620972	1.130971784	0.002892
NM_012008	370.2621766	1.9132185	-7.596401724	0
NM_012011	728.7298826	1.84874675	-8.62269273	0
NM_013559	3173.226227	7258.223005	1.193666005	0.000001
NM_013560	1272.114997	3790.124954	1.575016319	0.003403
NM_013602	5458.396642	11984.1906	1.134583335	0.044954
NM_013667	67.6544382	28.6270015	-1.240808015	0.00856
NM_013692	934.5714064	363.8706553	-1.36087919	0.000124
NM_013732	88.8964894	34.05263925	-1.384359828	0.007131
NM_013832	86.6078448	28.279901	-1.614720638	0.000002
NM_013847	1255.825232	2870.486535	1.192659584	0.000062
NM_013900	1375.231195	546.2960758	-1.331919211	0.000898
NM_015755	204.4925484	68.646957	-1.574780598	0.000186
NM_016854	105.7609046	41.54835275	-1.347943236	0.005322
NM_016869	21.4362634	54.127729	1.336314411	0.002614
NM_016974	1031.612509	2078.192087	1.010427837	0.008211
NM_017376	1807.977765	3959.306552	1.130870838	0.000029
NM_017391	26.5136762	56.80019275	1.099159107	0.002584
NM_018731	72.0707572	164.2041583	1.188004754	0.000004

NM_018863	773.2387472	298.8672483	-1.371409127	0.004317
NM_019723	67.5872926	33.1114595	-1.02942142	0.000385
NM_019754	81.5847566	33.92124	-1.266110713	0.000801
NM_019826	935.9960278	2220.717308	1.246451441	0.000424
NM_020002	830.3172068	358.1140768	-1.213243366	0.000005
NM_020013	11.8320134	66.23142075	2.484820215	0.013808
NM_020052	242.0996736	64.9576415	-1.898029977	0.000127
NM_020258	80.8506542	17.85575125	-2.178870613	0.000057
NM_020279	165.4116844	69.0471795	-1.260406759	0.002959
NM_020610	1152.198055	473.609888	-1.282617621	0.000001
NM_021347	1904.342364	429.3229645	-2.149157621	0.018234
NM_021390	45.7629918	102.6261183	1.165144665	0.002428
NM_021454	146.2493994	67.600107	-1.113333265	0.0001
NM_021704	33.1770952	113.7365635	1.777436637	0.000441
NM_022018	330.6276	133.7774898	-1.305371782	0.000084
NM_022024	109.9748116	50.895575	-1.111560995	0.004047
NM_023132	48.074961	140.429167	1.546485021	0.000006
NM_023184	32.9917494	73.4127865	1.153926084	0.004249
NM_023209	98.0310528	223.3585238	1.18805059	0.002608
NM_023224	62.2842668	27.3437315	-1.187657644	0.000204
NM_023395	140.0858222	67.9613595	-1.043524333	0.000327
NM_023707	6587.774104	416.7015473	-3.982704723	0.023885
NM_024204	53.1635058	131.686316	1.308597288	0.017294
NM_025404	453.4916272	198.3198908	-1.193246532	0.01067
NM_025549	733.8737182	173.582163	-2.079913125	0.000004
NM_025685	916.9947874	437.5283558	-1.067537013	0.00009
NM_026131	96.1708666	47.26815825	-1.024731266	0.000205
NM_026271	51.1578672	107.929037	1.077055033	0.001868
NM_026473	1230.950955	390.6782793	-1.655720329	0.001607
NM_026560	110.6980502	223.0737745	1.010891103	0.006314
NM_026619	576.7628884	153.7098058	-1.907769134	0.004191
NM_026770	4503.105653	2095.554265	-1.103588446	0.001118
NM_026778	267.4873522	131.0969978	-1.02883603	0.000963
NM_026785	155.3287932	355.18129	1.1932303	0.004836
NM_026853	812.1498324	1676.213731	1.045388298	0.007828
NM_026929	1715.850121	5694.250264	1.730582362	0.007953
NM_026935	149.1386728	461.2308448	1.628834588	0.00038
NM_027025	1063.79042	462.5552623	-1.201516308	0.000167
NM_027460	49.9465054	105.4497133	1.078099527	0.000214
NM_028145	107.6971004	44.17135575	-1.285796389	0.000018
NM_028183	426.9792252	201.2555893	-1.085137026	0.000024
NM_028386	48.0725218	120.7799315	1.329096368	0.00003
NM_028784	1262.116533	287.193981	-2.135747704	0.001158
NM_028894	38.4120424	88.97671475	1.211869156	0.014286
NM_028918	84.0556792	9.538194	-3.139557267	0.002101
NM_028955	66.805596	17.391193	-1.941612054	0.005424
NM_029238	116.3793904	43.13376125	-1.431946165	0.006069
NM_029285	34.4670254	74.8367285	1.118529697	0.001171
NM_029381	113.3695584	42.063556	-1.430390581	0.000001
NM_029614	1190.739833	413.1524233	-1.527112196	0.000616

NM_029723	24310.48403	8314.394575	-1.547895498	0.000046
NM_029796	79.323443	32.4045715	-1.291549942	0.047461
NM_030143	220.1375354	90.764852	-1.27819952	0.001106
NM_030209	419.2490548	137.011371	-1.613511899	0.012424
NM_030693	12008.74664	34215.57655	1.510567674	0.000008
NM_033042	81.7053062	203.1775778	1.314239519	0.003949
NM_033217	98.597502	46.77720775	-1.07574535	0.002643
NM_033601	530.7230612	233.403205	-1.185134867	0.001057
NM_033605	112.7791916	45.7685085	-1.301073723	0.000476
NM_053080	2638.050296	997.5915223	-1.402950961	0.036724
NM_053147	88.9747342	36.01708925	-1.304714124	0.000212
NM_053247	130.5038368	386.636152	1.566884319	0.000048
NM_133355	52.4072622	115.5833935	1.141095484	0.000647
NM_133851	55.390261	124.8338573	1.172305032	0.005915
NM_134022	433.8646862	86.798498	-2.321503182	0.004622
NM_134050	108.4471416	219.8439598	1.019487868	0.000195
NM_134090	1217.36536	571.0733708	-1.092014201	0.000027
NM_138686	192.932906	389.008589	1.011702783	0.002227
NM_145134	94.7588542	41.9572455	-1.175340786	0.009031
NM_145150	157.918661	343.2803193	1.120205486	0.010143
NM_145424	86.4518456	37.8161395	-1.192894672	0.001016
NM_145449	184.1920772	62.73032025	-1.553976174	0.005027
NM_145547	325.2659752	159.6645953	-1.026575477	0.000094
NM_145551	103.8069608	21.97427725	-2.240015567	0.000569
NM_146187	988.551963	392.0759635	-1.334183604	0.000183
NM_146879	36.1974988	106.8410038	1.561503518	0.000239
NM_153543	332.8249284	863.984936	1.376242664	0.000006
NM_153790	3382.463113	617.2910013	-2.454051536	0.000105
NM_172301	52.2320784	126.6955343	1.278357657	0.007397
NM_172659	1251.808427	439.4582065	-1.51021592	0.000024
NM_172665	87.5706344	41.776658	-1.067750077	0.000353
NM_172673	739.8538514	328.7146605	-1.170404512	0
NM_172921	60.5129604	23.8915905	-1.340741266	0.00003
NM_172951	72.4904936	23.79865775	-1.606911605	0.000032
NM_173739	216.650617	504.4092638	1.219224427	0.000004
NM_173762	168.1646676	378.8135238	1.171613218	0.005371
NM_174851	32.2923436	70.084399	1.117901184	0.00002
NM_174985	65.1718758	25.4835435	-1.354683621	0.000314
NM_175093	380.3325544	1290.604754	1.762713909	0.007004
NM_175138	794.4802552	358.0471983	-1.149861587	0.000044
NM_175149	1606.198913	720.8032898	-1.155973068	0.000019
NM_175293	142.5573792	59.1682855	-1.268646723	0.000004
NM_175362	76.3424908	25.53521825	-1.579997868	0.02102
NM_177049	199.052652	88.95544875	-1.161995211	0.000042
NM_177066	62.1487784	13.7812265	-2.173021743	0.000445
NM_177250	145.8834446	27.825557	-2.390333698	0.000232
NM_177292	70.384174	196.8410285	1.483707982	0
NM_177307	93.8947074	212.5727168	1.178840698	0.000092
NM_177577	272.3927056	131.1299338	-1.054691014	0.016025
NM_177645	26.1084802	53.612349	1.038046868	0.000014

NM_177920	917.9449784	291.2210645	-1.656292969	0.000004
NM_178751	344.3132168	158.8534258	-1.116025358	0.00011
NM_181407	423.8914136	202.0233903	-1.069172405	0.000468
NM_181748	45.7270504	101.6525215	1.152526235	0
NM_181860	23.3440206	54.53789675	1.224206002	0.047968
NM_182808	197.9690032	93.59913675	-1.08070743	0.002252
NM_182928	24.6285886	111.3752263	2.177022505	0.000842
NM_183183	160.1310274	59.4670145	-1.429091322	0.001545
NM_184088	48.560136	100.18545	1.044828633	0.000198
NM_194063	244.3058058	51.46144975	-2.247124143	0.000139
NM_201531	37.6124838	84.00615125	1.159283391	0.009185
NM_205823	90.4407018	44.166166	-1.034030588	0.001735
NM_207205	84.071669	40.684942	-1.04712478	0.001012
NM_207651	91.514345	39.92592825	-1.196671956	0.003207
NR_001463	2.1259342	474.9854915	7.803642692	0.000006
NR_001570	2.0373692	340.0523528	7.382905623	0.000017
NR_027980	139.3412512	56.05796225	-1.313631216	0.000001
NR_033207	241.0361966	118.0869258	-1.02940057	0.00016
NR_037956	189.4833292	458.5994103	1.275163575	0.003394
NR_037979	233.9830836	92.40255	-1.340399659	0.000981
NR_040619	155.1319984	72.67781275	-1.093909388	0.0003
NR_045098	521.050349	151.51317	-1.781979584	0.000015
NR_045424	73.8684568	5.0702935	-3.864817271	0.000016
XIST	2.4171114	3250.682315	10.39324289	0.000005
XM_003084492	254.663773	100.2293685	-1.345288446	0.000052
XM_003688767	1164.516817	552.4885365	-1.075715038	0.00079
